# Intra-Monozygotic Twin Pair Discordance and Longitudinal Variation of Whole-Genome Scale DNA Methylation in Adults

**DOI:** 10.1371/journal.pone.0135022

**Published:** 2015-08-06

**Authors:** Na Zhang, Shumin Zhao, Su-Hua Zhang, Jinzhong Chen, Daru Lu, Min Shen, Chengtao Li

**Affiliations:** 1 Shanghai Key Laboratory of Forensic Medicine, Institute of Forensic Sciences, Ministry of Justice, P.R. China, Shanghai, 200063, P.R. China; 2 State Key Laboratory of Genetic Engineering, Institute of Genetics, School of Life Sciences, Fudan University, Shanghai, 200433, P.R. China; Bellvitge Biomedical Research Institute (IDIBELL), SPAIN

## Abstract

Monozygotic twins share identical genomic DNA and are indistinguishable using conventional genetic markers. Increasing evidence indicates that monozygotic twins are epigenetically distinct, suggesting that a comparison between DNA methylation patterns might be useful to approach this forensic problem. However, the extent of epigenetic discordance between healthy adult monozygotic twins and the stability of CpG loci within the same individual over a short time span at the whole-genome scale are not well understood. Here, we used Infinium HumanMethylation450 Beadchips to compare DNA methylation profiles using blood collected from 10 pairs of monozygotic twins and 8 individuals sampled at 0, 3, 6, and 9 months. Using an effective and unbiased method for calling differentially methylated (DM) CpG sites, we showed that 0.087%–1.530% of the CpG sites exhibit differential methylation in monozygotic twin pairs. We further demonstrated that, on whole-genome level, there has been no significant epigenetic drift within the same individuals for up to 9 months, including one monozygotic twin pair. However, we did identify a subset of CpG sites that vary in DNA methylation over the 9-month period. The magnitude of the intra-pair or longitudinal methylation discordance of the CpG sites inside the CpG islands is greater than those outside the CpG islands. The CpG sites located on shores appear to be more suitable for distinguishing between MZ twins.

## Introduction

Monozygotic (MZ) twins have identical genomic DNA sequences, making it difficult for forensic scientists to distinguish between DNA samples from MZ twins using conventional chromosomal genetic markers [1]. Several studies used heterozygosity of mitochondria DNA to distinguishing between DNA samples [2].

In contrast to the relatively stable chromosomal DNA sequences, DNA methylation patterns are more dynamic due to genetic, environmental and stochastic factors throughout the life of an individual [3–9], providing a new possibility to distinguish between MZ twins.

Epigenetic discordance has been observed within MZ twin pairs both at specific loci [10–13], and across the genome [14, 15]. Most epigenetic studies in MZ twins have focused on common human diseases [10, 11, 15–18]. Recently, microarray-based analyses have revealed epigenetic differences between healthy juvenile MZ twins [14, 19–21]. In adult MZ twins, Boks et al. [22] measured DNA methylation at ~ 1500 CpG sites in whole blood samples using an array-based approach, and Gervin et al. investigated DNA methylation at 1760 sites in CD4^+^ lymphocytes using bisulfite sequencing [23]. At these specific loci, both studies identified extensive variations in DNA methylation between adult MZ twins, suggesting that MZ twins might be distinguishable based on their DNA methylation patterns. However, the extent whole-genome wide variation in DNA methylation patterns within healthy adult MZ twins is not well understood.

In forensic cases, suspects are usually arrested within weeks to months. A critical underpinning for using epigenetic markers for suspect identification is that DNA methylation patterns need to be stable for several months so that samples recovered from a crime scene can match samples collected from the arrested criminal. Thus, it should be carefully investigated whether longitudinal epigenetic variation in a span of months would affect the ability to distinguish between MZ twins.

Longitudinal epigenetic variations can be assessed using a cross-sectional approach [22, 24–26]. To date, only a few studies have estimated variation in methylation patterns within an individual over time at specific loci [9, 27, 28] or across the whole genome [15, 19, 21, 29, 30]. Although these longitudinal studies have demonstrated epigenetic drift on the time scale of years, to the best of our knowledge, no information is available regarding the degree of genome-scale methylation changes within healthy adult individuals within shorter intervals. Besides, such epigenetic drift within an individual has not been compared to DNA methylation discordance between MZ twins.

Here, we address three questions: (1) How are adult MZ twins different in terms of DNA methylation patterns? (2) How stable are DNA methylation over time? (3) Is the magnitude of epigenetic drift similar to, less than, or greater than the degree of intra-twin pair discordance?

To address these questions, we used the Illumina Infinium HumanMethylation450 (HM450) BeadChip platform to assess genome-wide DNA methylation profiles.; Blood samples from 10 healthy adult MZ twin pairs were used to assess the extent of intra-pair epigenetic differences. Furthermore, we tested whether genome-wide DNA methylation patterns of an individual drift within a time span of 3, 6, or 9 months in 8 individuals, including one MZ twin pair. A novel data analysis pipeline was developed by applying quantile normalization (QN) in lumi followed by beta-mixture quantile normalization (BMIQ) [31] on the raw data to correct for probe design bias and reduce any technical variability [32].

## Results

### Data acquisition and processing

**Fig 1:**
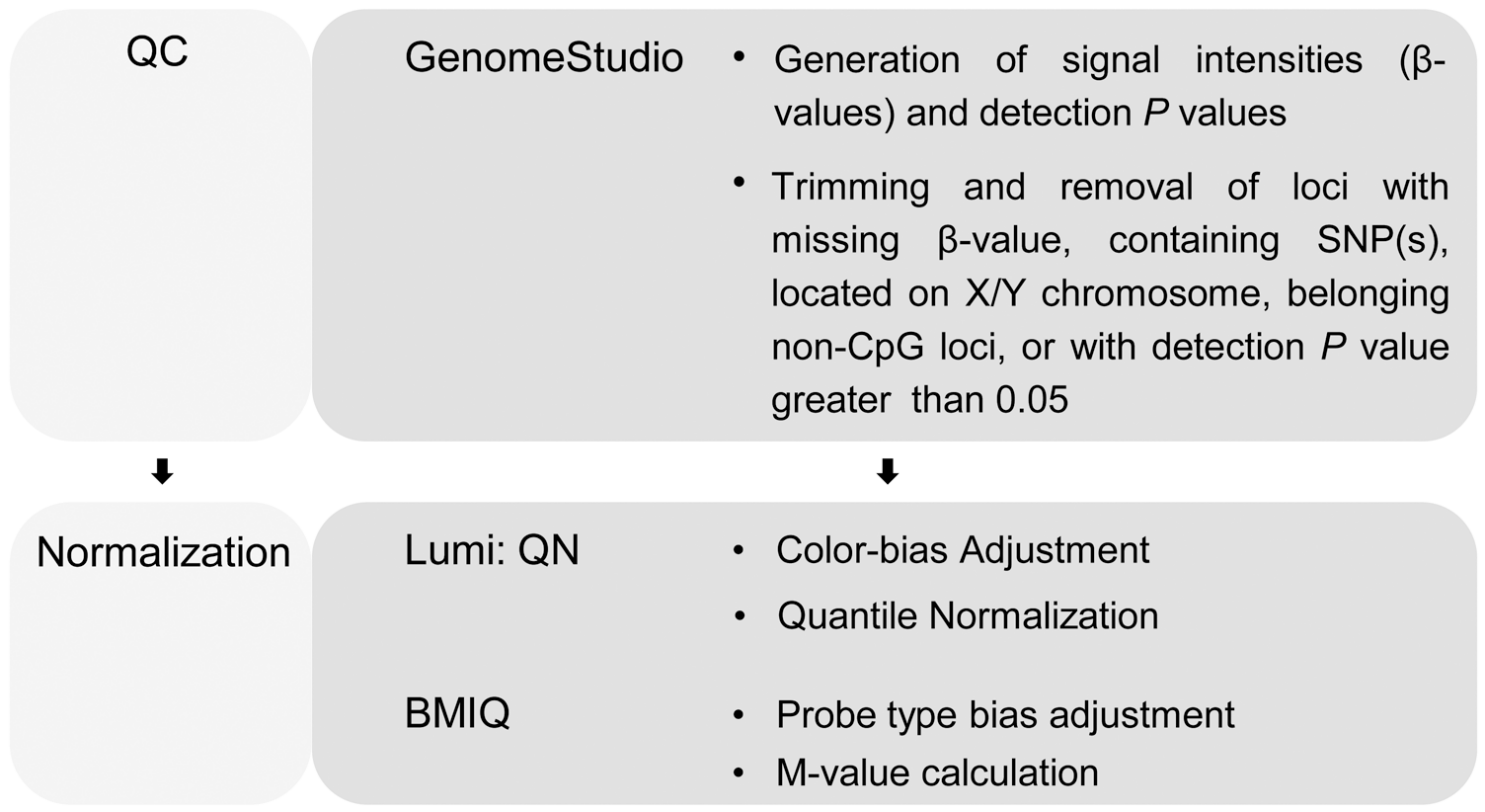
Workflow for DNA methylation analysis using Illumina Infinium HumanMethylation450 (HM450) BeadChip.

To assess the discordance in DNA methylation between MZ twins and within individuals over time on the whole-genome scale, whole blood from 10 pairs of MZ twins (Group A for MZ twins) and 8 individuals (including a pair of MZ twins) (Group B for longitudinal study) collected at 0, 3 (exception for Subject H), 6, and 9 months were processed using Illumina Infinium HM450 BeadChips (Table 1). Probes located on the X and Y chromosomes, probes containing SNP(s) or non-CpG loci, and probes with a detection *P* value exceeding 0.05 or missing β-values in any of the samples were removed from all individuals in the same group. This stringent strategy was implemented to minimize bias from sex-specific differences in methylation, low measurements due to SNP(s), and to control the number of probes per sample in each group. After excluding probes with potential bias, 375324 and 369187 unbiased CpG probes were selected and used for Groups A and B, respectively (S1 Table). The lumi-based QN+BMIQ was applied to the restricted datasets and β-values were transformed into M-values so as to improve performance in differential analysis of methylation levels [33] (Fig 1, also see Materials and Methods).

**Table 1:** Volunteer characteristics.

Group	# Volunteer	Age	Gender	Collection time points
**Group A**	MZ^a^ 1_A_, MZ 1_B_	31	F	
	MZ 2_A_, MZ 2_B_	25	M	
	MZ 3_A_, MZ 3_B_	45	M	
	MZ 4_A_, MZ 4_B_	74	M	
	MZ 5_A_, MZ 5_B_	54	M	
	MZ 6_A_, MZ 6_B_	43	F	
	MZ 7_A_, MZ 7_B_	41	M	
	MZ 8_A_, MZ 8_B_	45	F	
	MZ 9_A_, MZ 9_B_	23	F	
	MZ 10_A_, MZ 10_B_	32	M	
**Group B**	MZ 11_A_, MZ 11_B_	26	M	0, 3, 6, 9 m
	C	31	M	0, 3, 6, 9 m
	D	24	F	0, 3, 6, 9 m
	E	24	F	0, 3, 6, 9 m
	F	39	M	0, 3, 6, 9 m
	G	37	M	0, 3, 6, 9 m
	H	27	F	0, 6, 9 m

^a^: monozygotic.

### The reproducibility of the platform

**Fig 2:**
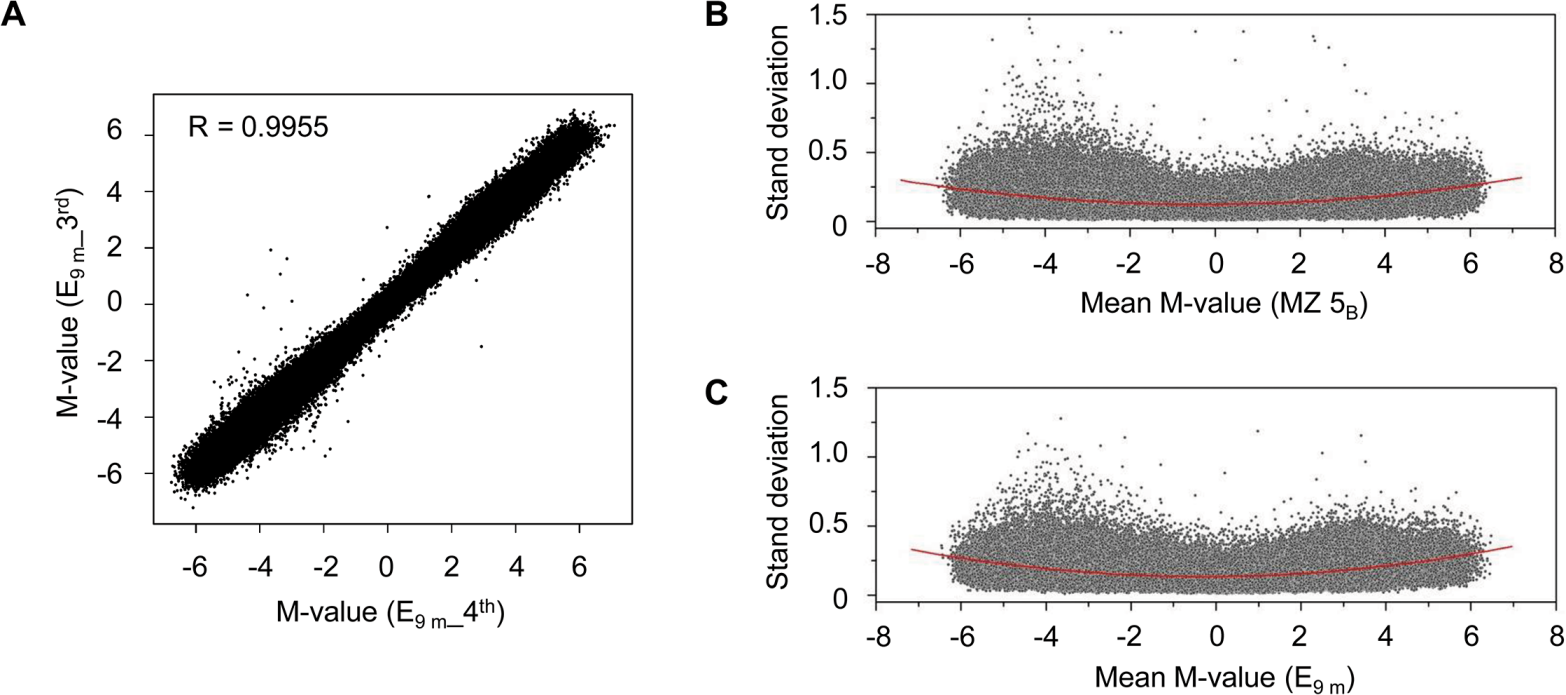
Technical reproducibility of the Infinium HumanMethylation450 (HM450) BeadChip platform. MZ 5_B_ from Group A and E_9 m_ from Group B were independently replicated 5 and 6 times on the Infinium HM450 BeadChip platform, respectively. (A) M-values for the 3^rd^ versus the 4^th^ replicate of E_9 m_ (R = 0.9955); (B and C) The standard deviation in M-values of 5 (B) or 6 (C) replicates of all 375,324 (B) or 369,187 (C) probes. The nonlinear fit is displayed as red lines, illustrating the trends in the SD for the M-values across the entire range.

To estimate the degree of technical variation, 5 and 6 sets of whole blood from two subjects (MZ 5_B_ from Group A, E_9 m_ from Group B) were independently processed using Infinium HM450 BeadChips, respectively. Technical replicates are highly correlated (Pearson correlation (R) ≥ 0.9967 in Group A run, R ≥ 0.9955 in Group B run (Fig 2A)), suggesting low technical variation and thus high reproducibility of the platform.

For each probe, the average M-value and the corresponding standard deviation (SD) were examined based on the technical replicates of MZ 5_B_ (Fig 2B) or E_9m_ (Fig 2C). Similar to a previous study [33], the SD remains approximately constant, indicating that the M-values for the DNA methylation levels are approximately homoscedastic. The SD of independent M-values validates that a constant difference threshold for M-value could be used to tabulate differentially methylated CpG sites. 996 (0.265%) and 1066 (0.289%) CpG sites generate SD larger than 0.5 in MZ 5_B_ and E_9 m_, respectively, while 126 (0.034%) and 93 (0.025%) CpG sites generate SD larger than 0.75 in MZ 5_B_ and E_9 m_, respectively. Consequently, a non-stringent down-limit methylation difference threshold was set at 1.0 (2 SD of 0.5) to minimize technical effects.

### Patterns of genome-wide differential DNA methylation in adult MZ twins

To assess variation in DNA methylation within MZ twin pairs, the DNA methylation level of 10 pairs of MZ twins (Group A) aged 23 to 74 years (Table 1) were measured on a genome-wide scale.

**Fig 3:**
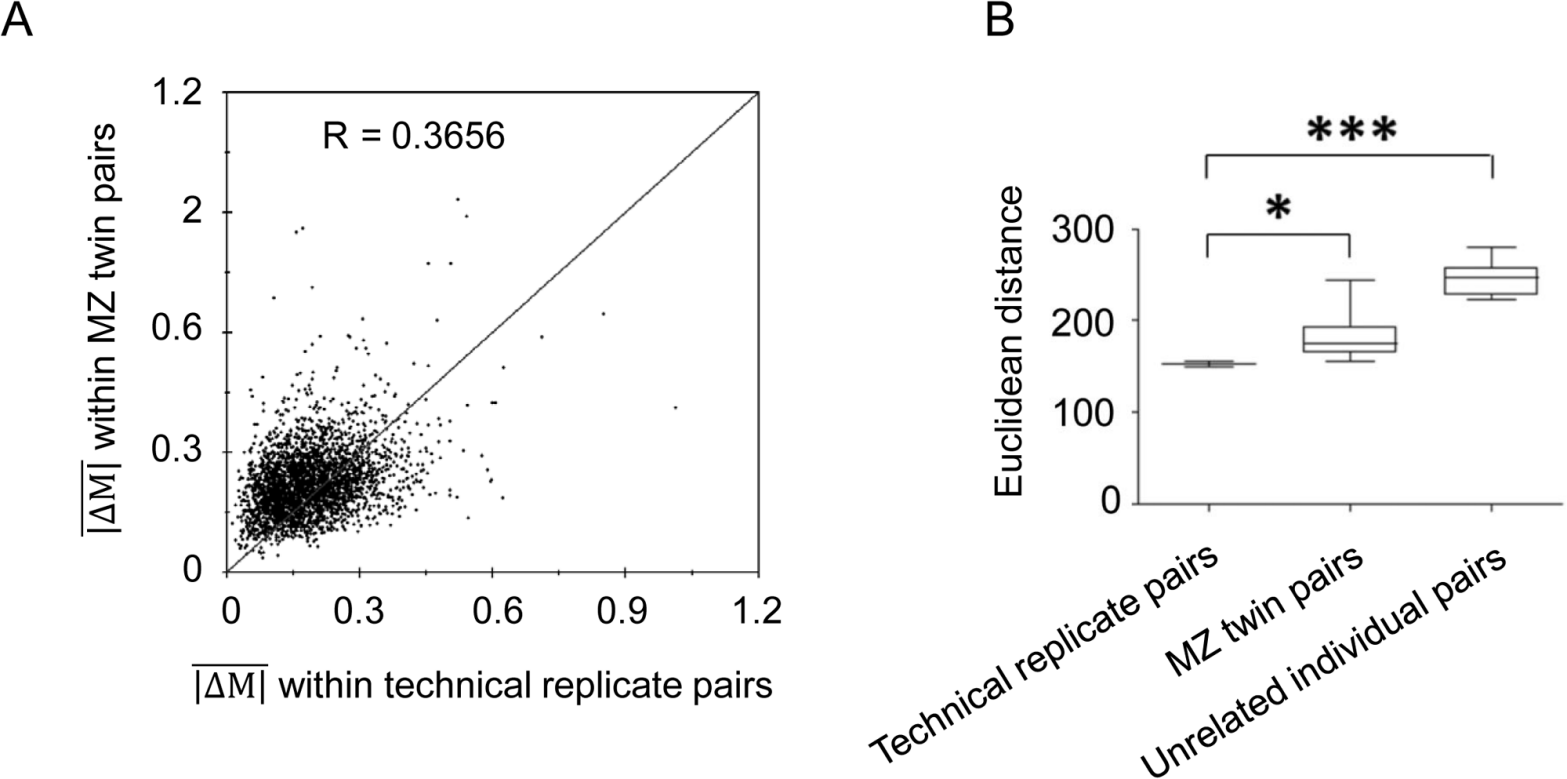
Examination of the variance between the monozygotic (MZ) twin co-pairs. (A) Mean of absolute difference of the M-values (|ΔM|) within MZ twin pairs plotted against technical replicate pairs at 375,324 CpG sites (R = 0.3656). (B) Distribution of the Euclidean distance calculated based on the M-values. From left to right: technical replicate pairs (N = 10, mean Euclidean distance = 152.3, based on the dataset containing independent 5 replicates from Subject MZ 5_B_), MZ twin pairs (N = 10, mean Euclidean distance = 182.3), and unrelated individual pairs (N = 11, mean Euclidean distance = 242.7). The black lines within each box represent the median of the Euclidean distance distribution. The boxes represent the inter-quartile range. The statistical analysis was performed using a non-parametric equivalent of one-way analysis of variance (ANOVA), the Kruskal-Wallis test, followed by a Bonferroni's/Dunn's multiple comparison test, *P < 0.05, ***P < 0.001.

Based on the M-values from 375,324 CpG sites, the Pearson correlation coefficients of the DNA methylation levels between the MZ twins were calculated. Correlations between each pair of MZ twins (mean R = 0.9955, R value of 0.9921 to 0.9968) are lower than among the replicates (mean R = 0.9969, R value of 0.9967 to 0.9970). Means of absolute difference between M-values (|ΔM|) within MZ twin pairs and the technical replicate pairs at the 375,324 CpG sites are displayed in Fig 3A (R = 0.3656). These data reveal that MZ twins have similar, yet distinct, DNA methylation profiles. The Euclidean distance calculated based on the M-values revealed significantly larger epigenetic dissimilarity within the MZ twin pairs relative to the technical replicate pairs at the whole genome level (Fig 3B). The unrelated individual pairs have even larger distance than the MZ twin pairs at the whole genome level. The Euclidean distance values are also moderately and positively associated with age (R = 0.7233, *P* = 0.0183) (S1 Fig).

### Determination of the DM CpG sites within MZ twin pairs

The CpG sites with a Benjamini and Hochberg False Discovery Rate (FDR)-adjusted *P* values below 0.05 and the absolute difference in the methylation level M-values (|ΔM|) values above 1.0 were considered significantly different in their methylation levels within MZ twin pairs.

The number of DM CpG sites between MZ twins that has |ΔM| > 1 and FDR-adjusted *P* < 0.05, 0.01, 0.001, and 0.0001is presented in Table 2. Most CpG sites display similar DNA methylation profiles, while a few (327 (0.087%) to 5,743 (1.530%) at *P* < 0.05 and 110 (0.029%) to 3,042 (0.81%) at *P* < 0.0001) CpG sites exhibiting dramatically different methylation levels within MZ twin pairs. These findings suggest that the difference in methylation within MZ pairs is subtle but significant.

**Table 2:** The number of differentially methylated (DM) CpG sites among the 375,324 filtered-in CpG sites between the monozygotic (MZ) co-twins with the dual thresholds of absolute difference in the M-value (ΔM) and the False Discovery Rate (FDR)-adjusted *P* value.

Threshold	#1 (%)	#2 (%)	#3 (%)	#4 (%)	#5^a^ (%)	#6 (%)	#7 (%)	#8 (%)	#9 (%)	#10 (%)
**|ΔM| > 1.0 & FDR-adjusted *P* value < 0.05**	1,460	1,074	513	5,743	3,481	327	906	922	509	452
	0.389%	0.286%	0.137%	1.530%	0.927%	0.087%	0.241%	0.246%	0.136%	0.120%
**|ΔM| > 1.0 & FDR-adjusted *P* value < 0.01**	1,191	804	369	4,707	2,971	236	692	738	377	347
	0.317%	0.214%	0.098%	1.254%	0.792%	0.063%	0.184%	0.197%	0.100%	0.092%
**|ΔM| > 1.0 & FDR-adjusted *P* value < 0.001**	934	579	249	3,713	2,415	164	489	555	262	233
	0.249%	0.154%	0.066%	0.989%	0.643%	0.044%	0.130%	0.148%	0.070%	0.062%
**|ΔM| > 1.0 & FDR-adjusted *P* value < 0.0001**	755	451	191	3,042	1972	110	399	455	188	171
	0.201%	0.120%	0.051%	0.810%	0.525%	0.029%	0.106%	0.121%	0.050%	0.046%

a: MZ 5A *vs*. MZ 5B_1^st^.

The numbers of DM CpG sites consistently identified in different twin pairs are presented in Table 3. No common DM CpG sites were identified in all 10 MZ twin pairs regardless of threshold values. Only 2, 2, 1 or 4 DM CpG sites were detected across 6, 6, 6, or 5 pairs using FDR thresholds of 0.05, 0.01, 0.001 or 0.0001, respectively (Table 3). Therefore, DM CpG sites between the twins are heterogeneous across the MZ twin pairs.

**Table 3:** The number of common differentially methylated (DM) CpG sites detected in 10 pairs of monozygotic (MZ) co-twins using the dual thresholds of absolute difference in the M-value (ΔM) and the False Discovery Rate (FDR)-adjusted *P* value.

Threshold	Total DM	No. of MZ twin pairs									
	CpG sites	1	2	3	4	5	6	7	8	9	10
**|ΔM| > 1.0 & FDR-adjusted *P* value < 0.05**	14,118	13,108	816	142	41	9	2	0	0	0	0
**|ΔM| > 1.0 & FDR-adjusted *P* value < 0.01**	11,504	10,764	598	105	30	5	2	0	0	0	0
**|ΔM| > 1.0 & FDR-adjusted *P* value < 0.001**	8,957	8,446	414	74	19	3	1	0	0	0	0
**|ΔM| > 1.0 & FDR-adjusted *P* value < 0.0001**	7,260	6,876	313	56	11	4	0	0	0	0	0

### Higher intra-pair discordance of methylation levels of DM CpG sites in CpG islands than that in other regions

**Fig 4:**
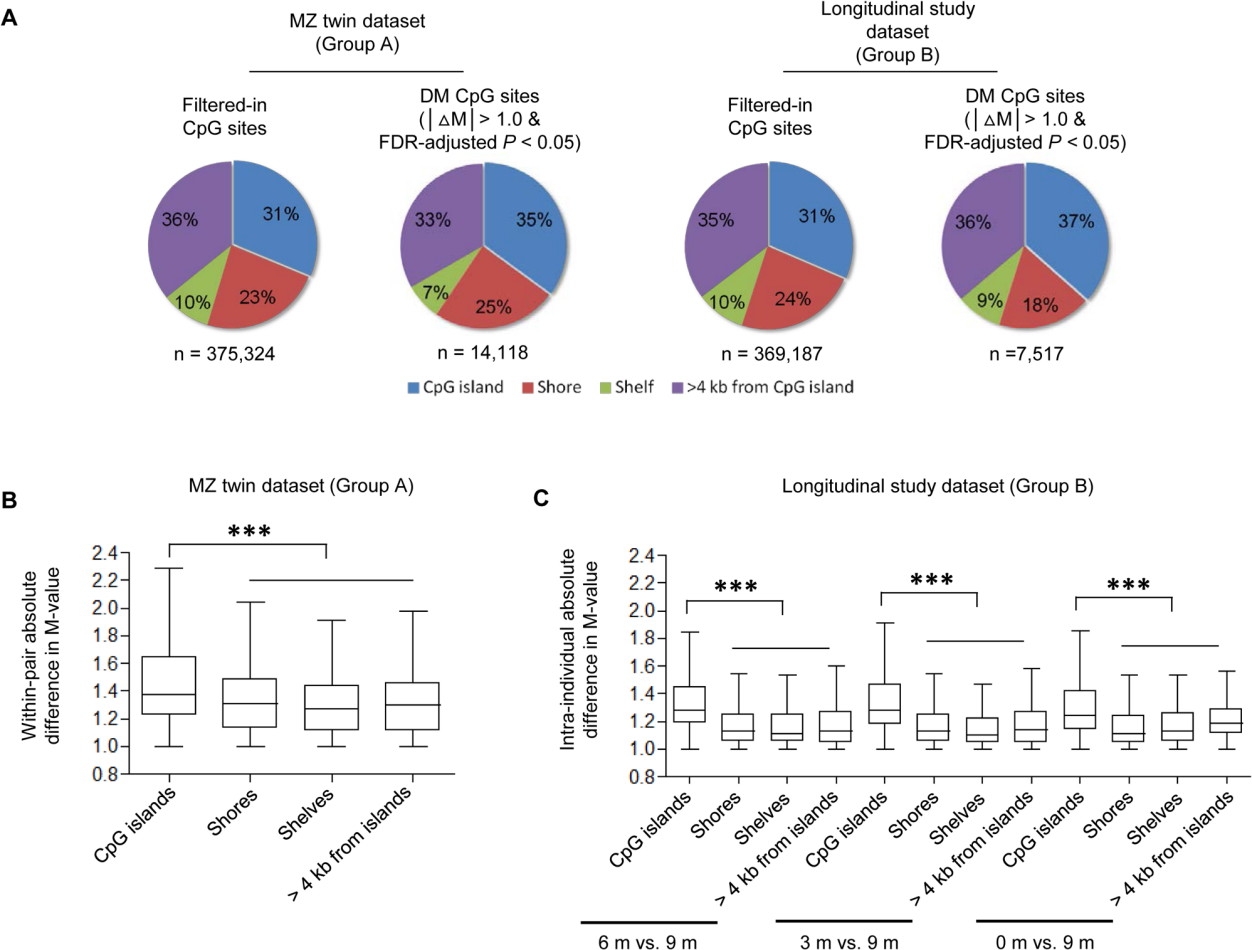
Relationship between position of CpG sites and differences in DNA methylation. (A) Comparison of the genomic distribution of DM CpG sites detected within at least one MZ twin pair (Group A, upper panel) or within a subject 3, 6, or 9 months apart (Group B, lower panel) under |ΔM| > 1 and FDR-adjusted *P* value < 0.05. n, the number of CpG sites satisfying the thresholds. (B-C) |ΔM| values within MZ twin pairs (B) or within the same individuals (C) plotted against their annotated locations. Black lines within each box represent the median of |ΔM|. The statistical analysis was performed using a non-parametric equivalent of one-way analysis of variance (ANOVA), the Kruskal-Wallis test, followed by a Bonferroni's/Dunn's multiple comparison test, ***P < 0.001.

The probes used in this study are designed to target UCSC CpG islands, shores (sequences up to 2 kb away from the CpG islands), shelves (sequences within 2 kb and 4 kb flanking CpG sites), or non-island regions (> 4 kb away from CpG islands) [34]. To test whether intra-pair DM sites appear more often in specific regions, we calculated the distribution of DM CpG sites in different Illumina-annotated genomic locations (Fig 4A, left panel). Most of the probes can be categorized into the respective Illumina-annotated CpG island class. Regardless of the cutoff values in |ΔM| and FDR-adjusted *P*, approximately 35% of the DM loci are annotated as CpG island probes, 25% as shore probes, 7% as shelf probes and 33% located in non-island regions (Fig 4A). To see if variations in methylation levels at the CpG sites correlate with their annotated genomic locations, we also compared |ΔM| at all DM CpG loci (|ΔM| > 1.0 and FDR-adjusted *P* < 0.05) in islands, shores, shelves, and non-island regions within the MZ twin pairs. Significantly higher values of |ΔM| are observed on CpG islands than in other regions (Fig 4B).

### Longitudinal changes in methylation patterns over 9 months

**Fig 5:**
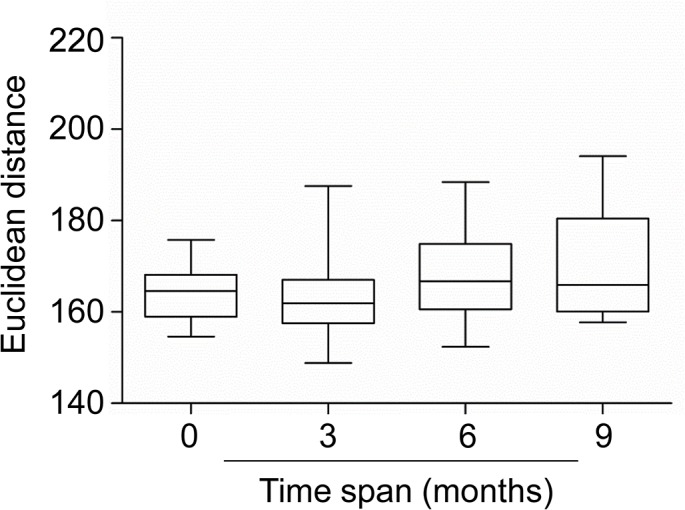
Distribution of euclidean distances between sample M-values. From left to right Euclidean distance between samples collected from the same individuals 0 month (N = 15 pairs, mean Euclidean distance = 163.9, based on the dataset containing independent 6 replicates from Subject E at 9 months), 3 months (0 months *vs*. 3 months, 3 months *vs*. 6 months, & 3 months *vs*. 9 months, N = 22 pairs, mean Euclidean distance = 163.9), 6 months (0 months *vs*. 6 months & 3 months *vs*. 9 months, N = 15 pairs, mean Euclidean distance = 168.8), and 9 months (0 months *vs*. 9 months, N = 8 pairs, mean Euclidean distance = 171.0) apart. Black lines within each box represent the median of the Euclidean distances. Boxes represent the inter-quartile range. The statistical analysis was performed using a non-parametric equivalent of one-way analysis of variance (ANOVA), the Kruskal-Wallis test, followed by a Bonferroni's/Dunn's multiple comparison test.

We measured genome-wide DNA methylation levels in 8 adults (24 to 39 years old), including a pair of MZ twins, sampled at 0, 3, 6 and 9 months (Table 1, Group B). 6 sets of whole blood samples from subject E at the 4^th^ time point (9 months) were collected and used to control technical variation. Based on Euclidean distance calculated from the M values of 369,187 CpG loci, at the whole genome level, longitudinal differences among samples collected from the same individual 3 (0 month *vs*. 3 months, 3 months *vs*. 6 months, and 6 months *vs*. 9 months), 6 (0 month *vs*. 6 months and 3 months *vs*. 9 months), or 9 (0 month *vs*. 9 months) months apart was not significantly larger than among samples collected at the same time from the same individual (based on the dataset containing independent 6 replicates from Subject E at 9 months) (Fig 5).

### Identification of longitudinal DM CpG sites

Despite genome level stability, CpG sites at specific loci exhibit significant longitudinal changes. To identify the DM CpG site within the same individuals over time, we compared the samples collected at 0, 3, and 6 months with that collected at 9 months.

The number of CpG sites that are longitudinal DM within the same individual detected under different threshold values are presented in Table 4. At |ΔM| > 1.0 and FDR-adjusted *P* value < 0.05, 99–395 (0.027%–0.107%) CpG sites are DM in the same individual 3 months apart, compared to 129–473 (0.035%–0.128%) DM CpG sites over 6 months and 148–1,514 (0.040%-0.410%) DM CpG sites over 9 months.

**Table 4:** The number of CpG sites with intra-individual longitudinal changes when using the dual thresholds of absolute difference in the M-value (ΔM) and the False Discovery Rate (FDR)-adjusted *P* value.

Threshold	Time span	MZ 11_A_	MZ 11_B_	C	D	E^#^	F	G	H
**|ΔM| > 1.0 & FDR-adjusted *P* value < 0.05**	3 months (6 m *vs*. 9 m)	309	357	395	355	99	352	295	306
	6 months (3 m *vs*. 9 m)	402	413	396	473	129	372	387	N/A*
	9 months (0 m *vs*. 9 m)	337	876	436	1,514	148	332	307	327
**|ΔM| > 1.0 & FDR-adjusted *P* value < 0.01**	3 months (6 m *vs*. 9 m)	232	257	289	260	58	249	216	224
	6 months (3 m *vs*. 9 m)	286	285	300	327	85	256	273	N/A
	9 months (0 m *vs*. 9 m)	230	600	322	1,175	96	229	218	239
**|ΔM| > 1.0 & FDR-adjusted *P* value < 0.001**	3 months (6 m *vs*. 9 m)	153	171	188	188	34	164	145	149
	6 months (3 m *vs*. 9 m)	187	191	209	207	56	183	177	N/A
	9 months (0 m *vs*. 9 m)	154	387	208	890	55	147	135	161
**|ΔM| > 1.0 & FDR-adjusted *P* value < 0.0001**	3 months (6 m *vs*. 9 m)	115	123	150	138	25	118	103	115
	6 months (3 m *vs*. 9 m)	125	148	150	137	37	129	126	N/A
	9 months (0 m *vs*. 9 m)	112	284	142	698	37	106	96	116

^#^: E_9 m__1^st^ used

*: not applicable.

A closer investigation on the DM CpG sites showed that most DM CpG sites are DM only during one of the three time spans (S2 Fig). This finding suggests that differences in methylation over this short time span are largely driven by stochastic factors.

Within all 8 individuals, DM CpG sites that are robustly detected during all 3 time spans are displayed in Table 5. Only 2 DM CpG sites are shared by all 8 individuals at low stringency (|ΔM| > 1.0 and FDR-adjusted *P* value < 0.05). The observed variation among individuals inferred that longitudinal changes in methylation patterns are likely caused by stochastic or unshared environmental factors.

**Table 5:** The number of common differentially methylated (DM) CpG sites detected across varying numbers of individuals up to 9 months apart using the dual thresholds of thresholds of absolute difference in the M-value (ΔM) and the False Discovery Rate (FDR)-adjusted *P* value.

Threshold	No. of individuals							
	1	2	3	4	5	6	7	8
**|ΔM| > 1.0 & FDR-adjusted *P* value < 0.05**	6,864	523	91	18	9	6	2	2
**|ΔM| > 1.0 & FDR-adjusted *P* value < 0.01**	5,000	350	60	11	5	6	2	2
**|ΔM| > 1.0 & FDR-adjusted *P* value < 0.001**	3,392	226	48	3	3	4	2	2
**|ΔM| > 1.0 & FDR-adjusted *P* value < 0.0001**	2,483	169	27	4	3	3	2	2

Among 7,517 significant DM CpG loci (|ΔM| > 1.0 and FDR-adjusted *P* value < 0.05), approximately 37% are in the CpG island, 18% were in the shores, 9% in the shelve, and 36% were located in the non-island regions (Fig 4A, right panel). And DM CpG loci located on CpG islands displayed a significantly greater degree of intra-individual discordance relative to those outside of CpG island regions (Fig 4C). This trend is consistent with the characteristic pattern of DM CpG sites in the MZ twin dataset (Fig 4B).

### Comparison of CpG sites exhibiting intra-pair discordance to those exhibiting longitudinal changes within the same individual

**Fig 6:**
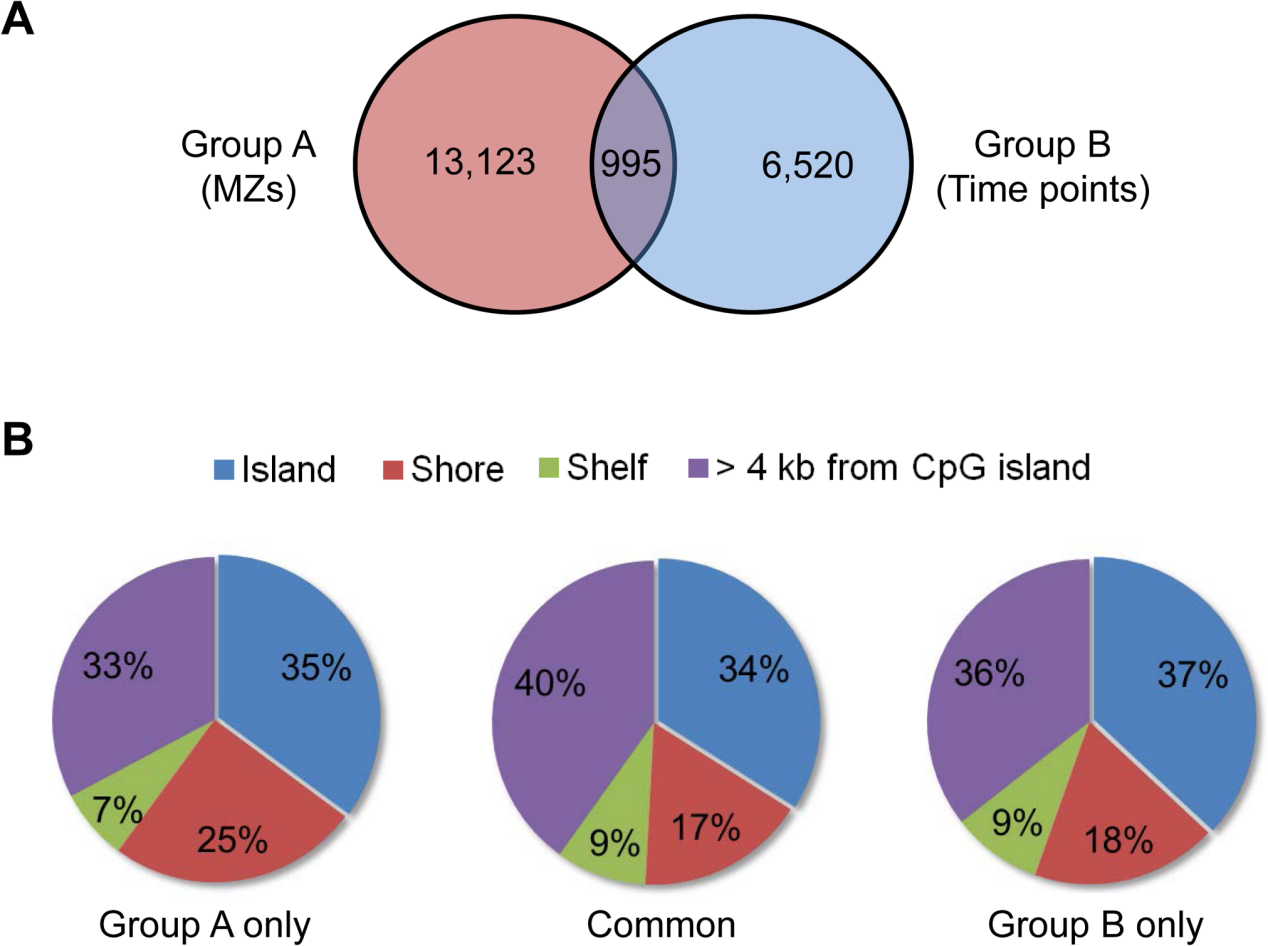
DM CpG sites detected in blood samples. (A) Pink circle: The numbers of DM CpG sites detected within 10 MZ twin pairs (Group A); blue circle: the numbers of DM CpG sites from 8 individuals collected at 4 (0, 3, 6, and 9 months) or 3 (0, 6, and 9 months) time points (Group B) Intersection (purple): The number of DM CpG sites detected in both groups (B) Comparison of the genomic distribution of DM CpG sites detected within MZ twin pair only, within individual over time only, and in both groups. DM CpG sites satisfy the difference criteria (|ΔM|) of 1.0 and FDR-adjusted *P* value of 0.05.

We compared the longitudinal DM CpG sites with the intra-MZ pair DM CpG sites identified under the dual thresholds of |ΔM| > 1 and FDR-adjusted *P* value < 0.05. Out of 14,118 DM CpG sites found within 10 MZ twin pairs, 995 overlapped with the 7,515 longitudinal DM CpG sites (Fig 6A). We then examined whether the intra-pair and longitudinal DM CpG sites have similar distributions. 25% of the intra-pair DM CpG sites are in the shore region, while only 18% of the longitudinal DM CpG sites and 17% of the DM CpG sites identified in both the intra-pair and longitudinal tests are in the shore region (Fig 6B). The finding indicated that the DM CpG sites located on shores within MZ twin pairs are more stable over time compared to those located at other regions in the genome.

### Clustering of longitudinal samples

**Fig 7:**
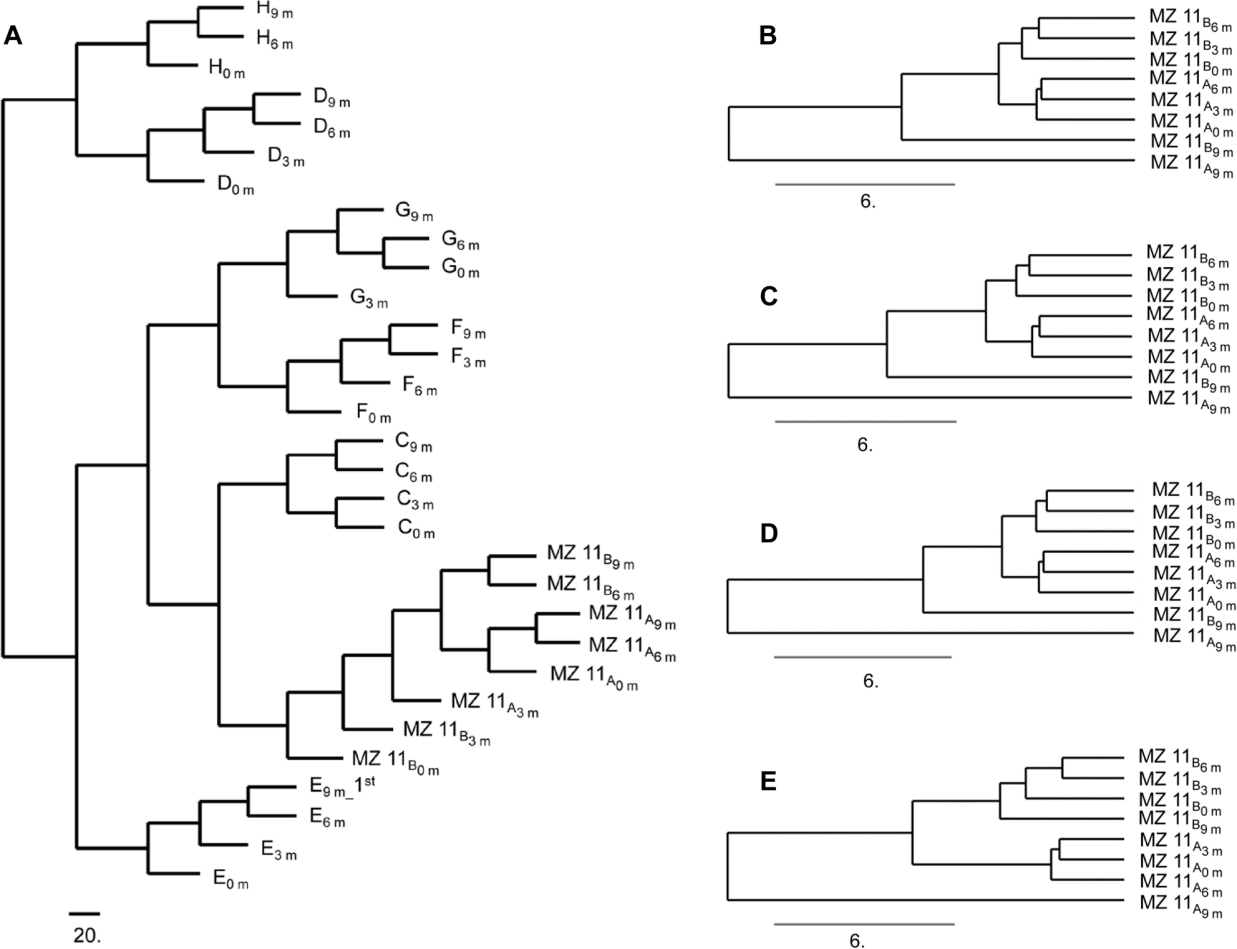
Similarity between individuals based on M-value. (A) Hierarchical clustering on 369,187 CpG sites from 31 whole blood samples collected from 8 individuals at 4 (0, 3, 6, and 9 months) or 3 (0, 6, and 9 months) time points. (B-D) Hierarchical clustering on CpG sites from 8 whole blood samples collected from MZ twin pair #11 at 0, 3, 6, and 9 months. The CpG sites satisfy |ΔM| > 1.0 and FDR-adjusted P value <0.05 within the MZ twin pair #11 with respect to sample collected at the 4^th^ visit (MZ #11_A_9m_ *vs*. MZ #11_B_9m_), and located across all regions (N = 453) (B), on the CpG islands (N = 228) (C), or on the shores (N = 78) (D). (E) Hierarchical clustering on the CpG sites from 8 whole blood samples collected from MZ twin pair #11 at 0, 3, 6, and 9 months. The CpG sites satisfy|ΔM| > 1.0 and FDR-adjusted P value <0.0001 within the MZ twin pair #11 with respect to samples collected at 4^th^ visit (MZ #11_A_9m_ *vs*. MZ #11_B_9m_), and located on the shores (N = 33).

To show similarity among DNA methylation patterns over time, unsupervised clustering of Group B dataset was performed using the M-values from 369,187 CpG loci (Fig 7A). For unrelated individuals, the samples from the same individuals collected at different time point cluster closer than samples from different individuals.

However, we observed close clustering of samples from the MZ #11 twin with the corresponding co-twin group (Fig 7A). The observation might due to the large proportion of CpG sites with concordant methylation patterns in the whole genome scale. 453 CpG loci, which were overlapping intra-pair DM sites at the dual thresholds of |ΔM| > 1 and FDR-adjusted *P* value < 0.05 within the MZ twin pair #11 at 4^th^ time point (MZ #11_A_9 m_ *vs*. MZ #11_B_9 m_), were selectively subjected to the 2^nd^ clustering algorithm using 8 samples from 4 time points. The resulting dendrogram also reveal a close clustering of MZ pair #11 (Fig 7B). After further excluding the probes located on neither CpG islands nor on shores, 228 (Fig 7C) and 78 (Fig 7D) out of 453 DM CpG loci were used to perform the 3^rd^ unsupervised cluster analysis, respectively. Those results also showed the misplacement of MZ #11 twin with the co-twin. Subsequently, we restricted this analysis to probes overlapping intra-pair DM CpG sites within MZ #11 co-pair at 4^th^ time point (MZ 11_A_9 m_ *vs*. MZ 11_B_9 m_) with most stringent thresholds (|ΔM| > 1 and FDR-adjusted *P* value < 0.0001). The result revealed that 8 samples from MZ pair #11 were correctively clustered when using the probes located on shores only ((Fig 7E, n = 33), which are more acceptable than that located across all regions in the genome (n = 153) or located on CpG islands only (n = 71) (Data not shown).

### Validation of identified DM CpG sites

To validate the identified DM CpG sites, we replicated 5 CpG sites (targeted by probes cg06188083, cg08122652, cg13304609, cg21549285, and cg26312951) by bisulfite pyrosequencing and Sanger sequencing in 8 DNA samples from MZ #11 collected at four time points (0, 3, 6, and 9 months). These five CpG sites exhibit the largest differences within MZ pair #11 (|ΔM| > 1 and FDR-adjusted *P* value < 0.0001) at each time point but did not exhibit longitudinal changes (0 month *vs*. 3 months, 3 months *vs*. 6 months, and 6 months *vs*. 9 months). The Infinium methylation M-values of these 5 sites from 8 samples were converted back to β-values, which might fall in one of three categories: hypomethylated (β-value of 0 to ≤ 0.2), heterogeneously methylated (β-value of > 0.2 to < 0.8), and hypermethylated (β-value of ≥ 0.8 to 1.0). Results from pyrosequencing are consistent with those given by Infinium HM450 BeadChip (S3 Fig), after the possibility of somatic mutations in the genomic DNA targeted by the CpG site probes was excluded with Sanger sequencing (Data not shown).

We next sought to determine the composition of blood cell types of samples to exclude differential methylation due to the variations in blood cell type compositions between samples. It has been shown that patterns of DNA methylation is robust among cell types [35–39]. In a recent study, leukocyte subsets were quantified using 34 CpG loci on Infinium HumanMethylation27 (HM27)/HM450 BeadChips [40]. Among the 34 CpG loci, 30 was involved in the MZ dataset in our study (Group A), and 29 was involved in the longitudinal dataset in our study (Group B). The cell type-specific DNA methylation patterns from the 30 or 29 CpG loci showed a significant consistency within MZ pairs #1–10 (S4A Fig), among samples from MZ 11_A_ or or MZ 11_B_ over different time spans (0 month *vs*. 9 months, 3 months *vs*. 9 months, and 6 months *vs*. 9 months) (S4B Fig) or within MZ #11 collected at the 4 time points (S4C Fig).Taken together, it was acceptable that the potential effect of somatic mutations or the variations of compositions of different white blood cell types on the determination of DM CpG sites was to the point of negligible.

Next, we aimed to perform the analysis in order to identify whether input DNA samples are completely converted by bisulfite or not. Recent work reported that three CpG loci (cg13107169 on *N4BP2*, cg16282679 on *EGFL8*, and cg16863382 on *CTRB1*) with stable hypermethylation across different human tissue types could be served as markers for evaluating the efficiency of bisulfite conversion [41]. We first investigated methylation levels at 3 CpG sites. And we also selected the 19 promoter-associated CpG sites located on CpG islands of 3 common housekeeping genes (*B2M*, *ACTB* and *GAPDH*) as markers for the efficiency of bisulfite conversion. The average M-value of methylation level at those 6 genes across 10 MZ pairs in MZ dataset or 8 individuals in longitudinal dataset are comparable and are all in line with the expectations, indicating that the bisulfite conversion efficiency was eligible in our HM450 assay (S5 Fig).

## Discussion

Distinguishing between MZ twins has great forensic importance. There are a number of forensic cases in which complete separation of samples from MZ twins would provide important probative evidence. Although MZ twins share the same genotype, they are not phenotypically identical. Numerous studies have revealed various epigenetic differences within MZ twin pairs with a primary focus on human diseases [10, 11, 15–18], young MZ twins [14, 19–21], or a small set of CpG sites [22, 23]. In this study, we used a whole genome array-based method to reveal methylation differences between adult MZ co-twins at ~ 450 thousand CpG sites throughout the genome. We also explored the variability of genomic methylation patterns in the same adult individual within a short time span.

### A reliable procedure for identifying DM CpG sites

The lumi:QN + BMIQ pipeline minimizes bias in probe design and batch effects [32] while maintaining sensitivity in detecting DM CpG sites. The lumi:QN + BMIQ pipeline facilitates more efficient detection of DM CpG sites than minfi:SWAN [19] or lumi-only [20] pipelines that were used in other recent studies in MZ using Illumina arrays. In this study, the nonlinear fitness of SD for the M-values exhibited a proximate homogeneity of variation (Fig 2B and 2C), similar with the result in a previous study [33].

Meanwhile, two other sources of bias should also be considered. The first one is the variations in blood cell type compositions, especially when the blood cell count corrections could not be performed for a blood spot in a forensic circumstance. The results indicated that the compositions of white blood cell types are very similar between MZ twins (S4 Fig). The second source of bias was insufficient or excessive bisulfite treatment. Results of the 3 CpG sites on hypermethylated genes (*N4BP2*, *EGFL8*, and *CTRB1*) and 19 promoter-associated CpG loci of 3 common hypomethylated housekeeping genes (*B2M*, *ACTB* and *GAPDH*) suggested that the bisulfite conversion efficiency was eligible in our HM450 assay (S5 Fig).

### Intra-pair and intra-individual discordance in DNA methylation

Our findings showed that there are discordance in DNA methylation patterns in a small proportions of CpG sites within MZ twin pairs, agreeing with earlier genome-scale studies on adult twins [22, 23]. These results indicated that DNA methylation patterns could potentially be utilized in distinguishing MZ twin from the matched co-twin. However, the DM CpG sites within each MZ twin pair is highly pair-specific (Table 3). No common DM CpG site was detected in all MZ twin pairs. Meanwhile, types of CpG sites that are DM over time in the same individual also vary greatly among individuals (Table 5). The intra-pair and intra-individual specific discordance in DNA methylation indicate that we cannot expect to distinguish between all MZ twins based on a common set of CpG sites.

Approximately 7% of DM CpG sites identified within MZ twin pairs overlap with longitudinal DM CpG sites (Fig 6A). In a real forensic case, because the suspect is usually arrested within months, such overlap could be confusing (Fig 7). An alternative strategy in data analysis was described by Feinberg and his colleagues, who annotated 227 variably methylated regions (VMRs) as relative stable, ambiguous or dynamic. They found that the dendogram based on clustering using the 119 stable VMRs was more reliable than that using the total VMRs [30].

Our data showed that |ΔM| of DM CpG sites located on CpG islands are significantly larger than other locations, both in the MZ twin study and in the longitudinal study (Fig 4B and 4C). This result is inconsistent with previous study using the HM27arrays in new born twins [20]. In that study, it was found that, at least in three different tissues, within-pair methylation discordance increases with the distance from the CpG sites to CpG islands in both newborn dizygotic (DZ) and MZ twin pairs. Also, shown in a later study, CpG sites showing developmental changes in DNA methylation tend to enrich in CpG shores and shelves [19]. Interestingly, the distribution of intra-pair discordance values in DNA methylation in twins was consistent across genomic location, while the result was consistent with their previous observation when only the probes present on the HM27 array were used to measure the within-pair discordance. We also observed that there are less longitudinal DM CpG sites in the shores (Fig 6B). This trend was in agreement with a recent DNA methylation study for subjects at an early age [21]. Wang and his colleagues assessed the longitudinal variation in DNA methylation patterns in infant cord and venous blood from birth to age 2 using the HM27 arrays [21]. Their findings indicated that most of the common DM CpG probes tend to target the shores. The discrepancies might be due to the differences in platform, sample size, data analysis methods, and especially the development stages (new born *vs*. adult) of subjects recruited in these studies.

Our study revealed that the intra-pair discordance in DNA methylation is positively associated with age (S1 Fig). Previous cross-sectional studies using low-resolution DNA methylation analyses [24] or array-based mRNA expression analyses also indicated that intra-pair discordance in DNA methylation increases with age [42]. It is a reasonable speculation that DNA methylation due to environmental factors accumulates over time and is much stronger in adults than in infants. Further, the “relatively longitudinally stable” DM CpG sites within MZ twin pair more likely locate on shores or shelves, rather than on CpG islands.

### Potential application of intra-pair DM CpG sites in distinguishing between MZ twins

It is axiomatic that samples taken from MZ twins at a same timepoint are distinguishable using intra-pair DM CpG sites. Nonetheless, in a real forensic case, the comparison is three-way: samples from both MZ twins need to be compared not only to each other but also to the sample taken from a crime scene weeks to months ago. Over this period of several months, environmental or stochastic factors could have altered the DNA methylation pattern in the twins.

On genome level, we observed no significant longitudinal alterations in the DNA methylation patterns in the same individual over a period of 3, 6, or 9 months. Therefore, most genomic regions do not undergo dynamic changes in methylation for up to 9 months in the same individual, consistent to the findings in a recent genome-wide study of DNA methylation in cord blood at birth and venous blood collected within the two years after birth using a Infinium HM27 array [21]. Similar results were also observed in adults using CHARM analysis on ~4.5 million CpG sites over a period of about 11 years [30]. On the other hand, we did identify a subset of CpG sites that were differentially methylated within individuals over the period of 9 months. These loci are more dynamic temporally and should not be used to distinguish between MZ twins. However, these temporally dynamic CpG sites are highly variable among individuals, therefore hard to predict *a priori*. As a result, it is unrealistic to establish a reliable list of temporally dynamic CpG site to be excluded from forensic tests.

Our clustering analysis showed no complete separation of samples from the MZ twin pair #11 collected at four different time points (Fig 7A). This result indicated that the magnitudes of the epigenetic differences within an individual over time are similar to those within MZ twin pair. The finding is consistent with a recent longitudinal study using an Infinium HM450 analysis on buccal epithelium collected from MZ and dizygotic twins which showed that most MZ twins cluster with their co-twins on age but not with the samples collected from the same individual at the different time points [19]. All of these findings point out the potential risks in attempting to distinguish between MZ twins based on intra-pair differential methylation given a background of longitudinal differential methylation.

However, the close clustering of samples from the MZ twin pair #11could have resulted from an inappropriate clustering strategy, because the dataset used contains more dynamic DM CpG sites located on CpG islands (Fig 7A–7C). When using the most stringent threshold (|ΔM| >1.0 & FDR-adjusted *P* value < 0.0001) together with relatively longitudinally stable DM CpG sites located on shores, the results of the clustering analysis is acceptable (Fig 7E). This result indicated that the DM CpG sites located on shores might be more effective in distinguishing between MZ twins.

In this study, we focused on peripheral blood samples. Several studies have revealed that tissue-specific DNA methylation patterns may arise from CpG loci on the control over differential expression of genes in different tissues [20, 43]. Future studies involving more tissue types may help to identify DM CpG sites that are more useful than to distinguish MZ twin samples collected from different tissue types.

This study contributes to the understanding of epigenetic differences between adult MZ twins and in the same person over time. Such knowledge is not only important in fundamental biological sciences, but also crucial for the development of tools in forensic sciences. The platform and analytical framework used in this study is also applicable to investigate the development mechanisms of complex disease.

## Materials and Methods

### Ethics statement

The human blood samples used in this study were collected with the approval of the Ethics Committee of the Institute of Forensic Sciences, Ministry of Justice, P.R. China. The samples were obtained from volunteers after receiving written informed consent. This study was approved by the Ethics Committee of the Institute of Forensic Sciences, Ministry of Justice, P.R. China.

### Subjects

11 monozygous twin pairs and 6 unrelated volunteers from China were recruited for the study with informed content (Table 1). All participants had no significant health problems or diseases according to their self-reported health records. Group A consisted of 10 pairs of MZ twins aged 23 to 74 years, including 8 female and 12 male subjects. Group B consisted of a pair of MZ (male) twins and 6 unrelated individuals (3 male, 3 female), aged 24 to 39 years. Except subject H, all participants in Group B were recalled every 3 months for 9 months (0, 3, 6, and 9 m). Subject H was studied only at 0, 6, and 9 months.

Four-milliliter EDTA-blood samples were collected at every visit and used for genome-wide analysis of DNA methylation. For MZ 5_B_ and E_9 m_, 5 and 6 independent whole blood samples were assessed for DNA methylation, respectively. Homozygosity of the twins was determined using 15 highly polymorphic short tandem-repeat loci with AmpF ℓ STR Identifier Kit (Applied Biosystems, Foster City, CA) [1].

### DNA extraction

Buffy coat was extracted from peripheral blood, followed by isolation of genomic DNA using the QIAamp DNA Blood Mini Kit (QIAGEN GmbH, Hilden, Germany) according to the manufacturer's instructions. All DNA samples were tested for degradation and purity using NanoDrop ND-1000 (Thermo Scientific, Waltham, MA, USA) and gel electrophoresis; any degraded or impure samples were excluded from the analysis.

### Genome-wide methylation analysis with Illumina Infinium HM450 BeadChips

The genome-wide DNA methylation profiles used in this study were generated using Illumina Infinium HM450 BeadChips (Illumina, San Diego, CA, USA). 1 μg of DNA extracted from whole blood was bisulfited using the EZ DNA Methylation-Gold kit (Zymo Research, Orange, CA) under the manufacturer’s standard protocol. 200 ng of bisulfite-treated DNA was amplified, enzymatically digested, and hybridized to the HM450 Beadchip containing 2 types of probes with different designs.

A total of 60 samples were processed using the HM450 BeadChips. DNA samples from the same MZ twin pairs and the same subjects were placed on the same BeadChip to minimize technical errors. The samples from the same group were processed in one run.

### Infinium methylation data extraction and processing

The data processing pipeline used in our study is shown in Fig 1. Raw microarray data containing signal intensities and detection *P* values were extracted using GenomeStudio (Illumina, San Diego, CA, USA) with no background subtraction or control normalization. The probes located on the X and Y chromosomes were removed to eliminate sex-specific differences during methylation. Probes containing SNP(s) sites were not used for further analysis because SNPs can lead to false negatives in detection of methylated sites. To ensure data quality, probes that failed to reach a detection *P* value of 0.05 or missing β-values in any of the samples were removed from all individuals in the group. The ch (non-CpG loci) probes were also removed before the analyses. Finally, 375,324 probes (out of 485,577) were used for Group A and 369,187 for Group B (S1 Table).

The HM450 platform contains 2 different bead types associated with 2 different chemical assays, InfiniumⅠand Infinium Ⅱ, which causes bias in probe design [44]. Unlike cancerous versus normal tissue, the difference in DNA methylation within a MZ twin pair or within an individual over a narrow time span might be subtle. Thus, proper algorithms need to be used to normalize the data from HM450 without sacrificing statistical power. We applied a quantile normalization (QN) in Bioconductor lumi package (version 2.12) followed by beta-mixture quantile normalization (BMIQ, version 1.0) [31] on the raw data to correct for probe design bias and reduce any technical variability [32]. Color-bias adjustment (Col.Adj) and QN were carried out on the signal intensities in the Bioconductor lumi package to control/minimize the batch effect in the experiment. Subsequently, the probe type-bias adjustment was performed on the β-values using BMIQ to correct the type 2 probe values, forming a distribution comparable to the type 1 probes [32]. Then, the Beta-values recommended by Illumina [45, 46] were converted to M-values [47] to improve the performance when assessing DM CpG sites in both highly methylated and unmethylated CpG sites [33]. The methylation level β-value and M–value statistics were calculated as follows [33]:

$$\beta =\frac{{\text{I}}_{\text{M}}}{{\text{I}}_{\text{M}}+{\text{I}}_{\text{U}}+\alpha }$$ (1)

$$\text{M}={\text{log}}_{2}\left(\frac{\beta }{1-\beta }\right)$$ (2)

where I_M_ and I_U_ represent the intensities measured for the methylated and unmethylated probes, respectively, and α is a constant.

Array data from this study have been submitted to the NCBI Gene Expression Omnibus (GEO) (http://www.ncbi.nlm.nih.gov/geo/) under the accession number GSE51388.

### Validation by quantitative bisulfite pyrosequencing and Sanger sequencing

We validated the microarray findings using quantitative bisulfite pyrosequencing. Primers used for pyrosequencing are listed in S2 Table. In brief, bisulfite conversion of 0.5 μg of fresh genomic DNA using the EpiTect bisulfite kit (QIAGEN) was followed by PCR amplification using the PyroMark PCR kit (QIAGEN). The PCR product was mixed with 4 pmol of the respective sequencing primer and streptavidin sepharose high-performance beads (GE Healthcare). The mixture was sequenced using PSQ 96 system (QIAGEN) with PyroMark Gold Q96 reagent kit (QIAGEN) following the manufacturer’s instructions. All PCRs and downstream steps were carried out in 3 replicates. The PyroMark CpG software 1.0.11 was used to analyze methylation status of CpG sites.

Simultaneously, Sanger sequencing was utilized to reveal whether or not the existence of somatic mutations in the genomic DNA region targeted by the CpG site probes validated with pyrosequencing, since somatic mutations would affect both probe behavior on the HM450 and pyrosequencing methylation detections. PCR amplification was performed with 20ng genomic DNA using the primers listed in S3 Table and AmpliTaq DNA Polymerase (Lifetechology) according to the standard PCR procedure for 35 cycles. All of the 5 primer pairs were designed with an optimum annealing temperature of 60°C. The PCR product was purified with the QIAquick PCR Purification Kit (QIAGEN), and then bi-directionally sequenced with BigDye Terminator v3.1 Cycle Sequencing Kit (Lifetechnology) using 3500 Genetic Analyzer (Lifetechnology).

### Statistical analysis

The Euclidean distances between samples were calculated using the M-values of all CpG sites that were measured. An unsupervised hierarchical clustering analysis was performed in R using package from the Bioconductor project [48]; Dendrograms were created using TreeDyn 198.3 [49, 50]. Pearson correlation analysis was performed with R software for comparisons of genome-scale methylation profiles within MZ pairs or among the technical replicates, or for comparisons of DNA methylation-based leukocyte quantification for different samples.

The significance of the differential methylation was evaluated using a chi-square allelic test taking the following steps:

- The SDs of M are obtained for each CpG sites based on the technical replicates in each group. The SDs are used to estimate systematic error.
- For a specific CpG site, a new statistic χ2is calculated as the following:
  $$\chi^{2}=\frac{{\left(\Delta M\right)}^{2}}{2SD^{2}}$$
      χ2 satisfies the chi-square distribution with one degree of freedom.
- For a specific pair, whole genomic control inflation factor (λ_gc_) is calculated as the ratio of the median of χ2 among all of the detected CpG sites with the theoretical median of chi-square distribution with one degree of freedom, the later approximately equals to 0.455.
- For a specific CpG site within MZ co-pairs or the same individual over time, χ2 is adjusted by λ_gc_ value, then *P* value for the differential methylation on the specific CpG site within pair is obtained according to the adjusted χ2.
- *P* value is adjusted with FDR approach to control false positives.

The CpG sites with |ΔM| exceeding 1.0 and FDR-adjusted *P* value below 0.05 were considered significantly DM.

### Validation of efficiency of bisulfite conversion

3 CpG sites on hypermethylated genes (cg13107169 on *N4BP2*, cg16282679 on *EGFL8*, and cg16863382 on *CTRB1*) [41] and 19 CpG sites on 3 housekeeping genes (cg00079638, cg00837838, cg07192821, cg08350173, cg19404757, cg19721944, and cg24134304 on CpG island of *B2M*; cg02356111, cg06003197, cg07476653, cg09041756, cg18080670, cg23162587, cg23175281, and cg23261233 on CpG island of *ACTB*; and cg00241355, cg09193981, cg09644986, and cg15869694 on CpG island of *GAPDH*) were used as markers for evaluating bisulfite conversion efficiency. The average methylation level is calculated at each gene according to the β-values given by Infinum HM450 BeadChip of CpG site(s) located on corresponding gene across 10 MZ pairs in MZ dataset or 8 individuals in longitudinal dataset, respectively.

## Supporting Information

S1 Fig Relationship between age and within-pair methylation discordance. Y axis, the differences within the monozygotic (MZ) twin pairs revealed by the Euclidean distance; x axis, age.
(TIF)

S2 Fig Longitudinal DM CpG sites 3, 6, and 9 months apart. DM CpG sites satisfy |ΔM| >1.0 and the FDR-adjusted *P* value < 0.05.
(TIF)

S3 Fig Cross-platform validation of DNA methylation. β-values given by Infinum HM450 BeadChip were plotted against percentage methylation given by bisulfite pyrosequencing for CpG loci of 8 samples from MZ #11 twins collected at 0, 3, 6, 9 months.
(TIF)

S4 Fig Comparisons of DNA methylation of CpG loci used for DNA methylation-based leukocyte quantification for different samples. (A) 30 CpG loci used to quantify leukocytes within each pair of samples from 10 pairs of MZ co-twin in Group A; (B and C) 29 CpG loci used to quantify leukocytes among samples collected from MZ 11_A_ or MZ 11_B_ at 0, 3, 6, and 9 months (B) and within sample paris from MZ #11 collected at same time point (C). Pearson correlation analysis revealed that the lowest *R* value was higher than 0.9487 (see S4 Table) and all of the *R* values were with significant *P* value lower than 0.0001.
(TIF)

S5 Fig Efficiency of bisulfite conversion. Average percentage methylation is shown at 3 hypermethylated genes (*N4BP2*, *EGFL8*, and *CTRB1*) and 3 housekeeping genes (*B2M*, *ACTB*, and *GAPDH*). The average methylation level (%) is calculated on β-values given by Infinum HM450 BeadChip of CpG site(s) on selected gene across 10 MZ pairs of Group A or 10 individuals at 4 time points (0, 3, 6, and 9 months) of Group B.
(TIF)

S1 Table Summary of the Infinium HumanMethylation450 (HM450) BeadChip probe statistics. (DOC)

S2 Table Bisulfite pyrosequencing primers. (DOC)

S3 Table Sanger sequencing primers. (DOC)

S4 Table Pearson *R* values of comparisons of DNA methylation-based leukocyte quantification for different samples. (DOC)
