# Supplementary figures and images for: Intra-Monozygotic Twin Pair Discordance and Longitudinal Variation of Whole-Genome Scale DNA Methylation in Adults
Source: PLoS One. 2015 Aug 6;10(8):e0135022. doi: 10.1371/journal.pone.0135022 (PMC4527769; doi:10.1371/journal.pone.0135022)

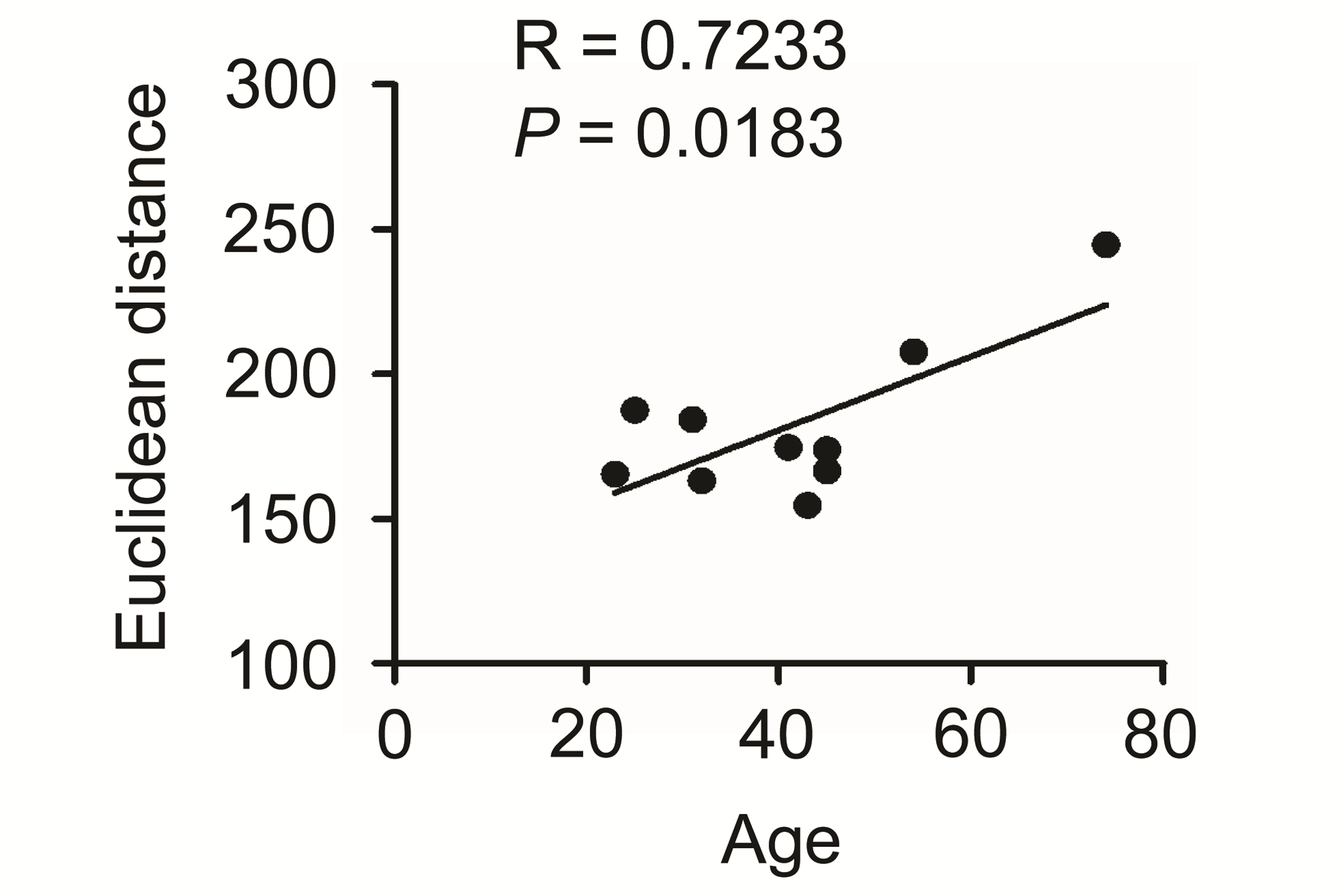

Supplement: S1 Fig — Y axis, the differences within the monozygotic (MZ) twin pairs revealed by the Euclidean distance; x axis, age. (TIF) [file pone.0135022.s001.tif]

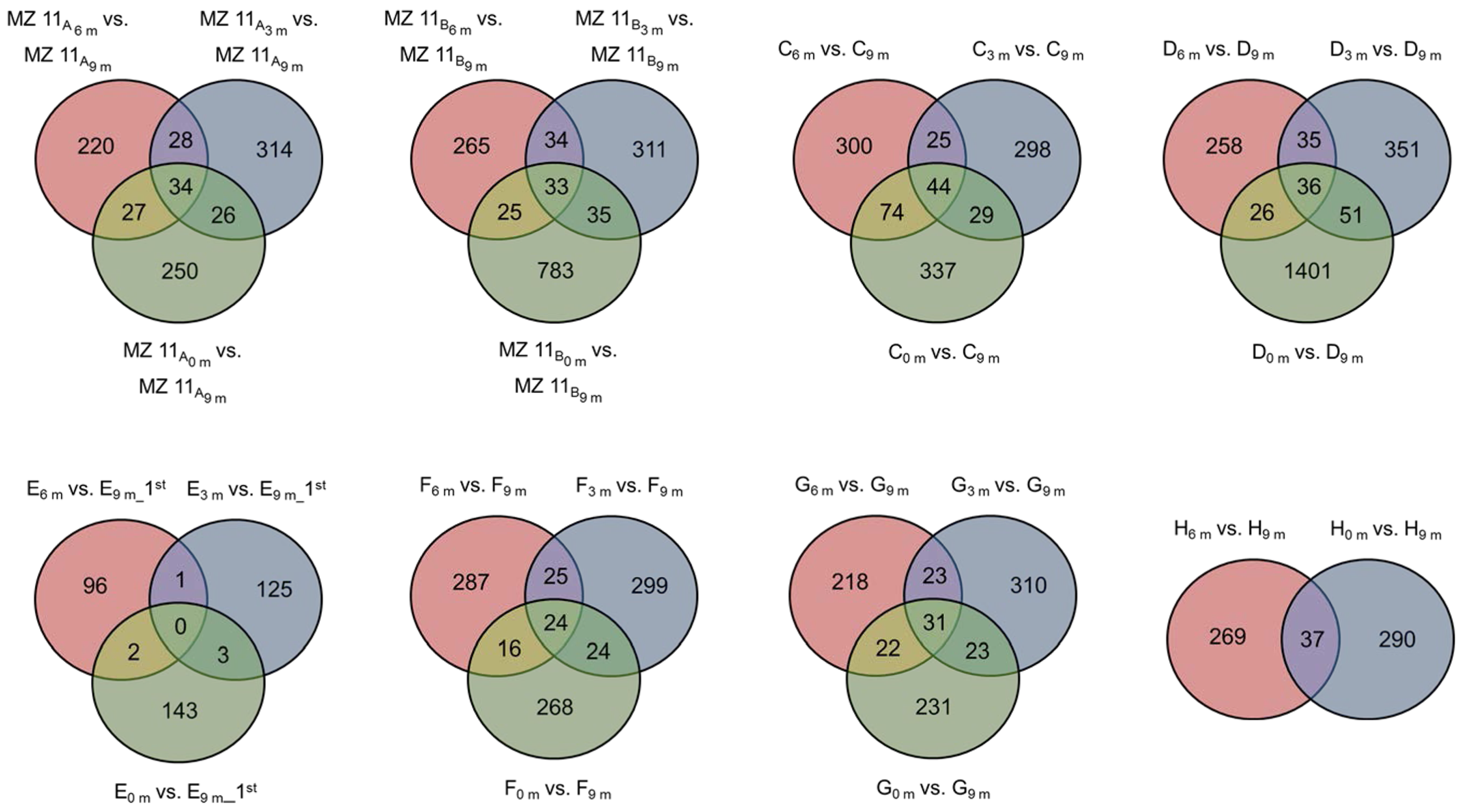

Supplement: S2 Fig — DM CpG sites satisfy |ΔM| >1.0 and the FDR-adjusted P value < 0.05. (TIF) [file pone.0135022.s002.tif]

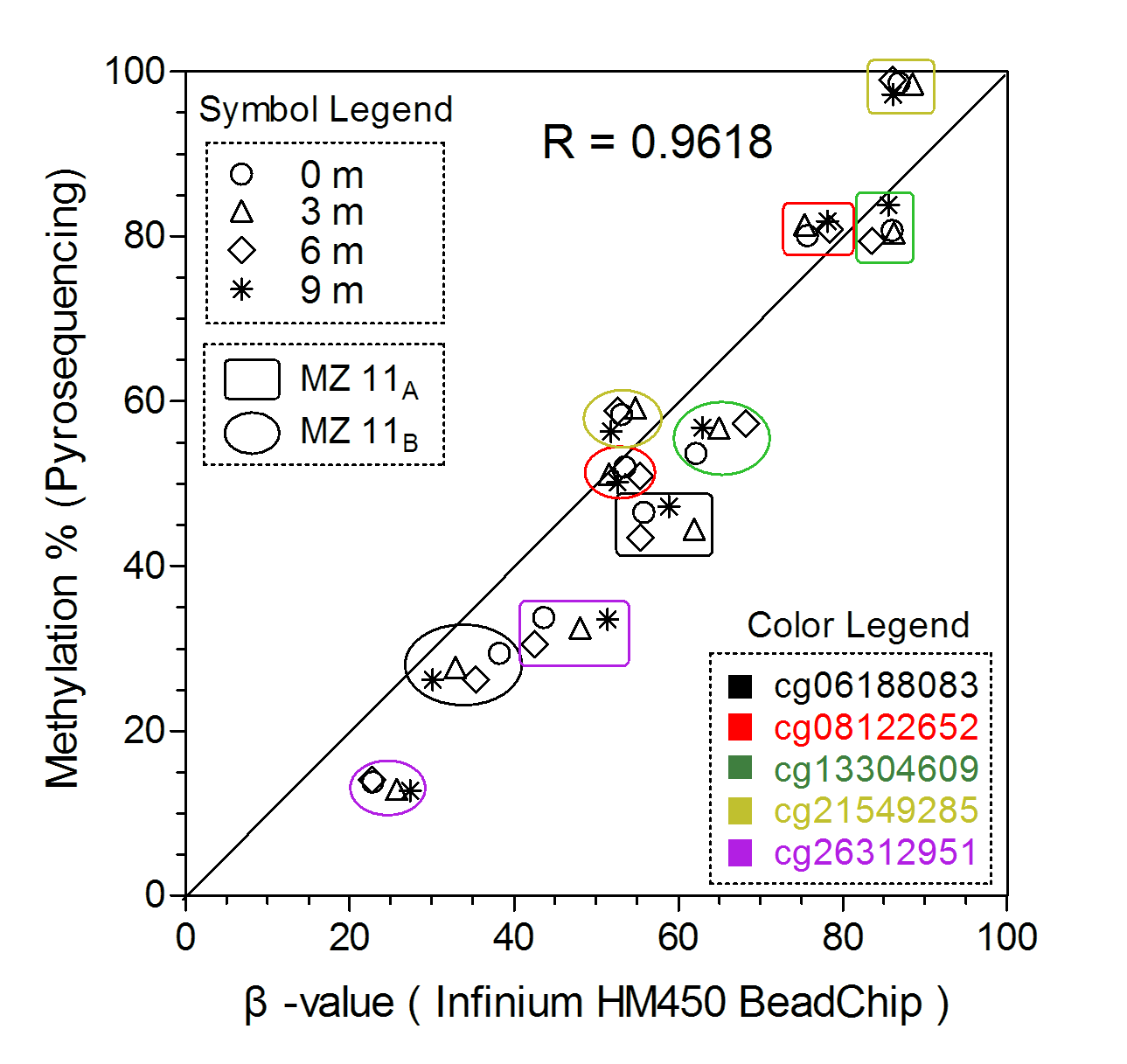

Supplement: S3 Fig — β-values given by Infinum HM450 BeadChip were plotted against percentage methylation given by bisulfite pyrosequencing for CpG loci of 8 samples from MZ #11 twins collected at 0, 3, 6, 9 months. (TIF) [file pone.0135022.s003.tif]

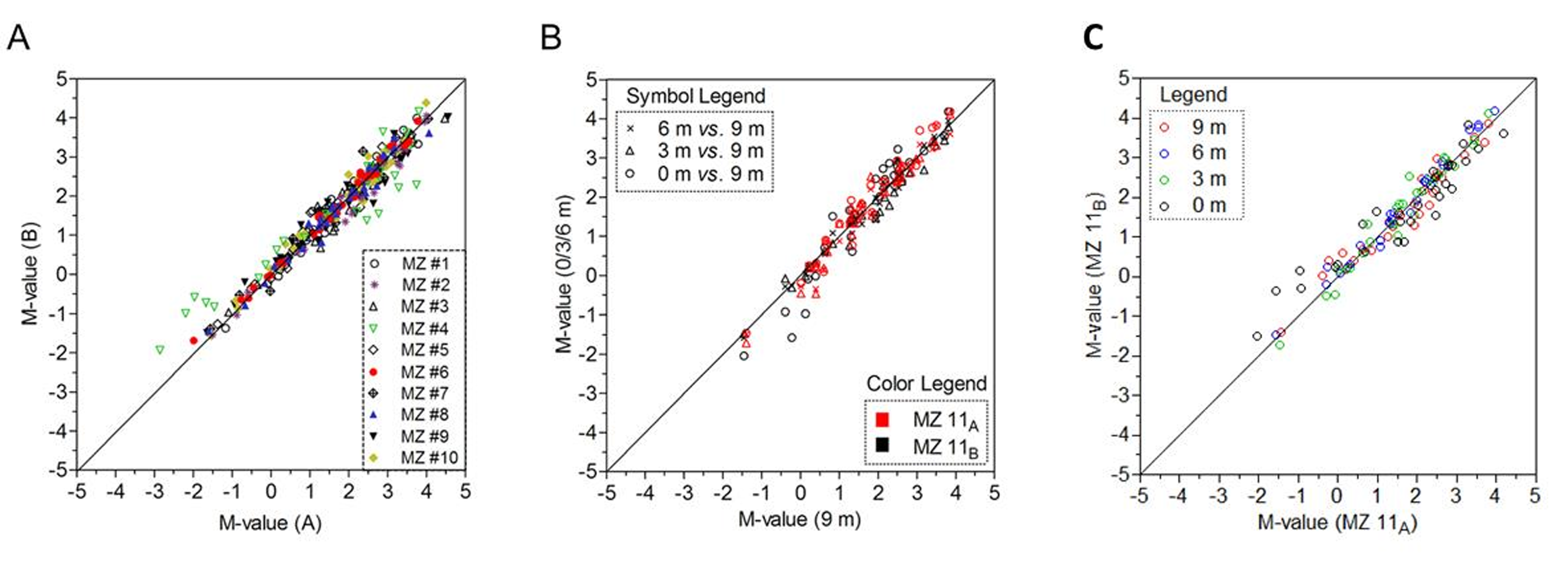

Supplement: S4 Fig — (A) 30 CpG loci used to quantify leukocytes within each pair of samples from 10 pairs of MZ co-twin in Group A; (B and C) 29 CpG loci used to quantify leukocytes among samples collected from MZ 11A or MZ 11B at 0, 3, 6, and 9 months (B) and within sample paris from MZ #11 collected at same time point (C). Pearson correlation analysis revealed that the lowest R value was higher than 0.9487 (see S4 Table) and all of the R values were with significant P value lower than 0.0001. (TIF) [file pone.0135022.s004.tif]

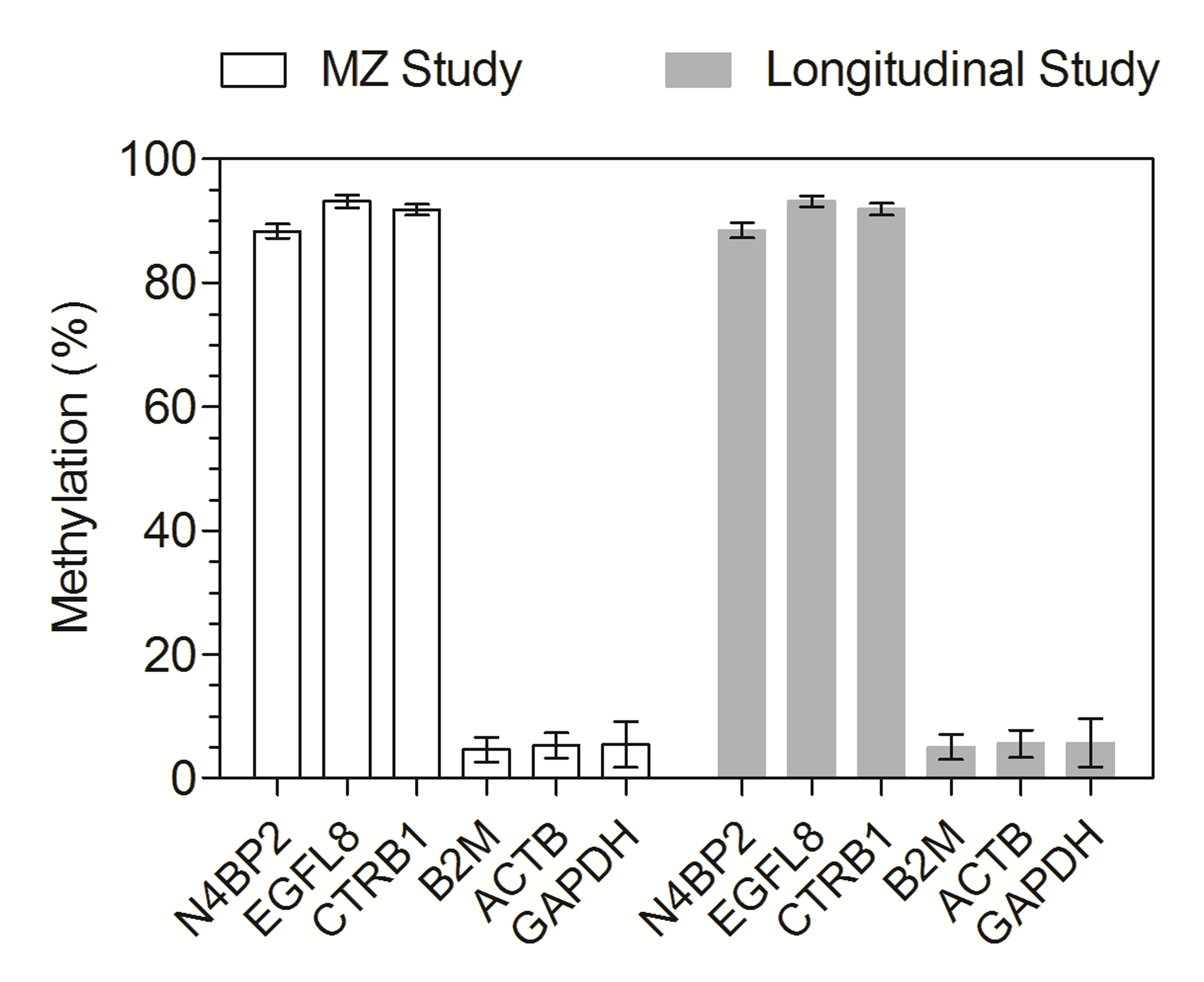

Supplement: S5 Fig — Average percentage methylation is shown at 3 hypermethylated genes (N4BP2, EGFL8, and CTRB1) and 3 housekeeping genes (B2M, ACTB, and GAPDH). The average methylation level (%) is calculated on β-values given by Infinum HM450 BeadChip of CpG site(s) on selected gene across 10 MZ pairs of Group A or 10 individuals at 4 time points (0, 3, 6, and 9 months) of Group B. (TIF) [file pone.0135022.s005.tif]
