# Supplementary material for: Intra-Monozygotic Twin Pair Discordance and Longitudinal Variation of Whole-Genome Scale DNA Methylation in Adults
Source: PLoS One. 2015 Aug 6;10(8):e0135022. doi: 10.1371/journal.pone.0135022 (PMC4527769; doi:10.1371/journal.pone.0135022)
Supplement: S1 Table — (DOC) [file pone.0135022.s006.doc]

**S6 Table. Summary of the Infinium HumanMethylation450 (HM450) BeadChip probe statistics.**

|  | **MZ^a^ twin dataset (%)** | **Longitudinal study dataset (%)** |
| --- | --- | --- |
| **Total loci** | 485,577 (100.0) | 485,577 (100.0) |
| **Loci containing SNP(s)^b^, non-CpG loci, or on Chr X/Y** | 102,736 (21.2) | 102,736 (21.2) |
| **Loci with missing β-value or detection p value > 0.05** | 6,617 (1.4) | 12,754 (2.6) |
| **Filtered-in loci** | 375,324 (77.3) | 369,187 (76.0) |

^a^: monozygotic;

^b^: single nucleotide polymorphism
