# Supplementary material for: Intra-Monozygotic Twin Pair Discordance and Longitudinal Variation of Whole-Genome Scale DNA Methylation in Adults
Source: PLoS One. 2015 Aug 6;10(8):e0135022. doi: 10.1371/journal.pone.0135022 (PMC4527769; doi:10.1371/journal.pone.0135022)
Supplement: S2 Table — (DOC) [file pone.0135022.s007.doc]

**S7 Table. Bisulfite pyrosequencing primers.**

| **Infinium probe ID** | **Primer** | **Sequence (5’-3’)** | **Sequence to Analyze** |
| --- | --- | --- | --- |
| **cg06188083** | Forward primer: | GATTAATGAATAGGGAGAAGGTGTTGTTTG | TTTTG**Y**GAGTGGTTTGAATTATATTAATTT GA |
|  | Reverse primer: | Biotin-AATTTCCTCCTTCACATTTACACACA |  |
|  | Sequencing primer: | TGTTTGTAGTTTTTTAAATTGTG |  |
| **cg08122652** | Forward primer: | GAGGATATATTTATAAGTAGGGATGAGT | G**Y**GGTAGAGAGGTTTGAGATAAGGTTTT |
|  | Reverse primer: | Biotin-CCAACCATATACAAAAACCTTATCTC |  |
|  | Sequencing primer: | TTATAAGTAGGGATGAGTG |  |
| **cg13304609** | Forward primer: | GGTTTGTTTGGAATGGAGTGA | TTATAAGYGGTGGGGTTAGGGAGGTGA**Y**GA GGGTGTAAGAGGGTTTTGTAAGTT |
|  | Reverse primer: | Biotin-TAAAAACTTACAAAACCCTCTTACAC |  |
|  | Sequencing primer: | GGAGTGATAGTATTGGAT |  |
| **cg21549285** | Forward primer: | TGGGAAGTAGAGTTTAGTGATGTT | T**Y**GTAGTTGTYGGTTAGGAATTTTAGTG |
|  | Reverse primer: | Biotin-CCCCCCAAACCCTACTAATAT |  |
|  | Sequencing primer: | TGTGTTATTTTTTAGTTTTGGA |  |
| **cg26312951** | Forward primer: | TGAGTTTTGGGAGGGAATTG | TGTTTTTAAAATYGA**Y**GGGGGGAAGGATAT GTTTAGGTTTAA |
|  | Reverse primer: | Biotin-AACCCCAACCCAAACCTACTAAAT |  |
|  | Sequencing primer: | GAAGATTTTTAATTATTAATGTATT |  |

Y(bold): the corresponding CpG locus in the Infinium probe
