# Supplementary material for: Intra-Monozygotic Twin Pair Discordance and Longitudinal Variation of Whole-Genome Scale DNA Methylation in Adults
Source: PLoS One. 2015 Aug 6;10(8):e0135022. doi: 10.1371/journal.pone.0135022 (PMC4527769; doi:10.1371/journal.pone.0135022)
Supplement: S3 Table — (DOC) [file pone.0135022.s008.doc]

**S8 Table. Sanger** sequencing primers.

| **Infinium probe ID** | **Primer ^a^** | **Sequence (5’-3’)** | **Amplicon length (bp) ^b^** | **Physical postins in Hg19 ^c^** |
| --- | --- | --- | --- | --- |
| **cg06188083** | Forward primer: | GGGATATGTCTACTTGAGTTGTCCT | 276 | chr10:91,092,893-91,093,168 |
|  | Reverse primer: | TCTGTAAGCCATTCAGTATGCCTAT |  |  |
| **cg08122652** | Forward primer: | TGGGACAAATTCCTAGAAATGGA | 291 | chr3:122,281,791-122,282,081 |
|  | Reverse primer: | GTGAAAAGCCTTGAAGAGCAGTG |  |  |
| **cg13304609** | Forward primer: | TCAATTTTGATATGCCATTCCTC | 349 | chr1:79,085,055-79,085,403 |
|  | Reverse primer: | CAACTGAATTACTGGGCTAAGGA |  |  |
| **cg21549285** | Forward primer: | TGGGAAGTAGAGTCCAGTGATGC | 212 | chr21:42,799,039-42,799,250 |
|  | Reverse primer: | CAAATGCACTGCTACCTACCAAA |  |  |
| **cg26312951** | Forward primer: | CTGGACGGAGTTCGGGACA | 272 | chr21:42,797,703-42,797,974 |
|  | Reverse primer: | GGCCTGCTGAATCAGATGCTC |  |  |

^a^: All of the primer pairs were designed with optimum annealing temperature of 60℃;

^b^: Bi-directional Sanger sequencing for each amplicon with corresponding PCR primers;

^c^: Physical positions of each amplicon in human genome obtained with the In-Silico PCR of UCSC Genome Browser on Human Feb. 2009 (GRCh37/hg19) Assembly (http://genome.ucsc.edu/).
