# Supplementary material for: Intra-Monozygotic Twin Pair Discordance and Longitudinal Variation of Whole-Genome Scale DNA Methylation in Adults
Source: PLoS One. 2015 Aug 6;10(8):e0135022. doi: 10.1371/journal.pone.0135022 (PMC4527769; doi:10.1371/journal.pone.0135022)
Supplement: S4 Table — (DOC) [file pone.0135022.s009.doc]

**S9 Table. Pearson *R* value of comparisons of DNA methylation-based leukocyte quantification within different sample pair.**

| **MZ Study ^a^** | | **Longitudinal Study ^a^** | |
| --- | --- | --- | --- |
| **MZ #1** | 0.9830 | **6 m *vs.* 9 m (MZ 11_A_)** | 0.9797 |
| **MZ #2** | 0.9882 | **3 m *vs.* 9 m (MZ 11_A_)** | 0.9754 |
| **MZ #3** | 0.9765 | **0 m *vs.* 9 m (MZ 11_A_)** | 0.9831 |
| **MZ #4** | 0.9495 | **6 m *vs.* 9 m (MZ 11_B_)** | 0.989 |
| **MZ #5** | 0.9843 | **3 m *vs.* 9 m (MZ 11_B_)** | 0.9824 |
| **MZ #6** | 0.9945 | **0 m *vs.* 9 m (MZ 11_B_)** | 0.9622 |
| **MZ #7** | 0.9758 | **MZ 11_A_ *vs.* 11_B_ (9 m)** | 0.9752 |
| **MZ #8** | 0.9860 | **MZ 11_A_ *vs.* 11_B_ (6 m)** | 0.9912 |
| **MZ #9** | 0.9739 | **MZ 11_A_ *vs.* 11_B_ (3 m)** | 0.9807 |
| **MZ #10** | 0.9835 | **MZ 11_A_ *vs.* 11_B_ (0 m)** | 0.9487 |

^a^: all of the *R* values with significant *P* value lower than 0.0001.
